# Association between XRCC3 rs861539 Polymorphism and the Risk of Ovarian Cancer: Meta-Analysis and Trial Sequential Analysis

**DOI:** 10.1155/2022/3915402

**Published:** 2022-08-08

**Authors:** Siya Hu, Yunnan Jing, Fangyuan Liu, Fengjuan Han

**Affiliations:** ^1^Department of Obstetrics and Gynecology, Heilongjiang University of Chinese Medicine, Harbin 150040, China; ^2^Department of Acupuncture and Moxibustion, Heilongjiang University of Chinese Medicine, Harbin 150040, China; ^3^Department of Obstetrics and Gynecology, The First Affiliated Hospital of Heilongjiang University of Chinese Medicine, Harbin 150040, China

## Abstract

**Background:**

Current studies on the relationship between XRCC3 rs861539 polymorphism and ovarian cancer risk have been inconsistent. Therefore, we performed a meta-analysis to explore their association.

**Methods:**

Six electronic databases (PubMed, Embase, Web of Science, Cochrane Library, China National Knowledge Infrastructure, and China Wanfang Database) were searched for relevant studies published before December 2021. Meta-analysis, subgroup analysis, sensitivity analysis, and publication bias analysis were performed using Stata software 16.0. Trial sequential analysis (TSA) was performed using TSA 0.9.5.10 Beta software.

**Results:**

A total of 12 studies were included in 9 literatures, comprising 4,634 cases of ovarian cancer and 7,381 controls. After Bonferroni correction, the meta-analysis showed an association between XRCC3 rs861539 polymorphism and ovarian cancer risk in the heterozygote model and the dominant model (GA vs. GG: OR = 0.88, 95%CI = 0.81-0.96, *P* = 0.003; GG vs. GA+AA: OR = 0.89, 95%CI = 0.82-0.96, *P* = 0.004). In an ethnically stratified subgroup analysis, XRCC3 rs861539 was shown to reduce the risk of ovarian cancer in Caucasian in the heterozygote model and the dominant model (GA vs. GG: OR = 0.88, 95%CI = 0.81-0.96, *P* = 0.004; GG vs. GA+AA: OR = 0.88, 95%CI = 0.81-0.96, *P* = 0.004). In the control source and detection method stratified subgroup analysis, hospital-based studies and PCR-RFLP-based studies were found to increase ovarian cancer risk (GG vs. AA: OR = 1.30, 95%CI = 1.05-1.62, *P* = 0.016; GG vs. AA: OR = 1.31, 95%CI = 1.06-1.62, *P* = 0.013).

**Conclusion:**

This meta-analysis showed a significant association between XRCC3 rs861539 polymorphism and ovarian cancer risk, especially in Caucasians. Large-scale multicenter case-control studies in more different regions will be needed in the future.

## 1. Introduction

Ovarian cancer is the most lethal of the gynecologic malignancies, with 314,000 new cases and 207,000 deaths worldwide in 2020, and its morbidity and mortality are increasing year by year [1, 2]. Ovarian cancer is not a single disease; it can be divided into at least five different histological subtypes with different biological and molecular properties. Moreover, the onset and progression of ovarian cancer are insidious, usually diagnosed late and difficult to treat [3, 4]. Therefore, it is of great significance to identify genes associated with high risk of ovarian cancer.

DNA damage caused by endogenous (e.g., chemical modifications and replication errors) and exogenous (e.g., ultraviolet radiation, ionizing radiation, and various chemicals) factors can give rise to genomic instability and somatic mutations [5, 6]. The accumulation of abnormal somatic mutations can trigger malignant cell transformation [7], which is a crucial cause of carcinogenesis and malignant tumor progression. Of all the DNA damage, DNA double-strand breaks (DSBs) are the most detrimental because there is no intact template strand to ensure accurate repair [7]. DNA double-strand break repair (DSBR) includes homologous recombination (HR) and nonhomologous terminal junction (NHEJ), in which HR is more accurate [7]. X-ray repair cross-complementing 3 (XRCC3) encodes a member of the RecA/RAD51-related protein family and is involved in homologous recombination repair (HRR) of DNA DSBs [8] and cross-link repair [9, 10]. A common polymorphism of the XRCC3 gene, SNP rs861539, is a C-to-T amino acid substitution at codon 241 in exon 7 of XRCC3, which may affect the function of the enzyme and/or its interaction with other proteins involved in DNA damage and repair [11]. XRCC3 polymorphism has been linked to the risk of multiple cancers, such as breast cancer [12, 13], thyroid cancer [13, 14], melanoma skin cancer [15], and lung cancer [16, 17].

Although many studies have explored the association between XRCC3 rs861539 polymorphism and ovarian cancer risk [18–26], the conclusions have been inconsistent. There have also been meta-analyses of the association between XRCC2 polymorphism and ovarian cancer risk [27, 28], but the results need to be supplemented. To obtain more accurate results, more comprehensive studies were included, and meta-analysis was performed to assess the association of XRCC3 rs861539 polymorphisms with ovarian cancer risk.

## 2. Materials and Methods

### 2.1. Search Strategy

To identify eligible case-control studies, we conducted a comprehensive search across six databases (PubMed, Embase, Web of Science, Cochrane Library, China National Knowledge Infrastructure, and China Wanfang Database) for studies published up to December 2021. We searched the databases using combinations of the following keywords: “XRCC3 or X-ray repair cross-complementing 3 or Thr241Met or rs861539 or 18067C/T” and “ovarian cancer” and “polymorphism or polymorphisms.” Additional articles were searched by checking the references of relevant studies.

### 2.2. Selection Criteria

The studies included in this meta-analysis need to meet the following criteria: (1) focusing on the association of XRCC3 polymorphism rs861539 with ovarian cancer risk, (2) case-control studies, and (3) providing sufficient information on genotype frequency in the case and control groups. The main reasons for exclusion were as follows: (1) reviews, cell or animal studies, and meta-analyses; (2) information on genotype frequency is unclear or absent; and (3) studies contained duplicate data.

### 2.3. Data Extraction

Two reviewers independently screened the literature according to the selection criteria, extracted essential data from the included studies, and then cross-checked to reach a consensus. The essential data we extracted included the first author's name, year of publication, country, ethnicity, total number of cases and controls, genotype frequency of the case group and the control group, and value of Hardy-Weinberg equilibrium (HWE). If an article had a case-control series of studies, we treated each study as an individual study in this meta-analysis.

### 2.4. Quality Assessment

The quality of the included studies was assessed using the Newcastle-Ottawa scale [29], which is divided into three dimensions, including selection, comparability and exposure. Studies with a maximum score of 9 on the scale and a score of less than 5 will be excluded.

### 2.5. Statistical Analysis

We assessed the association between XRCC3 rs861539 polymorphism and ovarian cancer risk using odds ratios (ORs) and 95% confidence intervals (CIs) in five genetic models, including homozygote model (GG vs. AA), heterozygote model (GA vs. GG), dominant model (GA+AA vs. GG), recessive model (GG+GA vs. AA), and additive model (G vs. A) [13, 30]. Bonferroni correction was applied to avoid multiple testing error, and *P* < 0.01 (0.05/5) was considered statistically significant. Chi-square-based *Q* statistic test and *I*^2^ value were used to test heterogeneity between studies. When *P* < 0.1 or *I*^2^ > 50%, significant interstudy heterogeneity was indicated, and random effect model was used for analysis; otherwise, fixed effect model was used instead [31]. Subgroup analyses based on ethnicity, control source, and detection method were performed to explore specific associations [32]. Sensitivity analysis was carried out by removing one article at a time. We evaluated publication bias using funnel plots, Begg's test, and Egger's test. The funnel plot is to observe whether the two sides are symmetrical, and the other two depend on the size of *P* value. *P* < 0.05 indicates that the meta-analysis may have publication bias. The analysis for the present study was completed by Stata software 16.0 (StataCorp, College Station, TX, USA).

### 2.6. Trial Sequential Analysis

Meta-analysis may lead to false-positive and false-negative results due to random error and lack of statistical accuracy [33]. We conducted TSA to estimate the required information size (RIS) and the reliability of the results based on 5% risk of type I error (*α* = 0.05, two sided), 80% power of study (*β* = 0.20), and a case-control event proportion calculated from meta-analysis [34]. TSA was performed using TSA 0.9.5.10 Beta software (Copenhagen Trial Unit, Denmark).

## 3. Results

### 3.1. Literature Search and Study Characteristics

**Figure 1:**
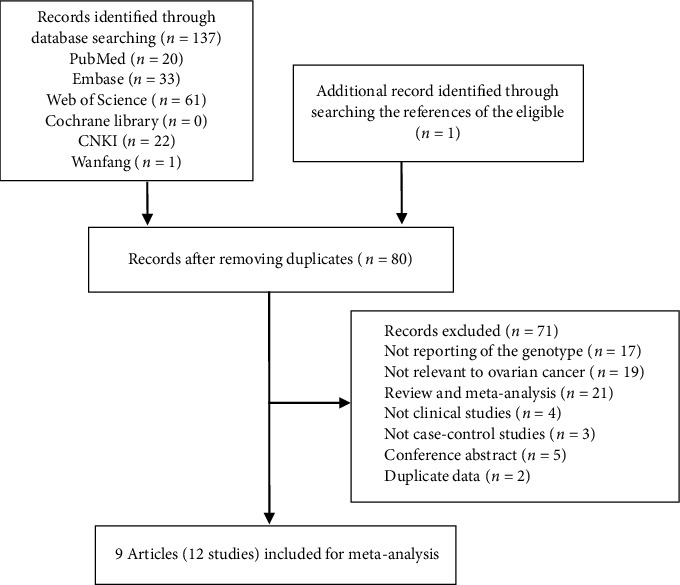
Flowchart of literature selection process.

According to the search strategy, we found 137 articles in the database (20 in PubMed, 33 in Embase, 61 in Web of Science, 22 in China National Knowledge Infrastructure, and 1 in China Wanfang Database). And 1 article was identified through searching the references of the eligible. Of these, 129 articles were further excluded due to duplicates (58), no reporting of the genotype (17), unrelated to ovarian cancer (19), reviews and meta-analyses (21), nonclinical studies (4), non-case-control studies (3), conference abstracts (5), and duplicate data (2). Ultimately, 9 articles with 12 studies were included for meta-analysis [18–26]. The flowchart of the literature selection process is shown in Figure 1.

The 12 studies included 4,634 cases of ovarian cancer and 7,381 controls, with 10 studies conducted in Caucasians [18–21, 24–26], one in Asians [22], and one in a mixed population [23]. The main information for each study is presented in Table 1. The distribution of all control genotypes included in the study conformed to HWE (*P* > 0.05).

### 3.2. Meta-Analysis and Subgroup Analyses

The meta-analysis results of XRCC3 rs861539 polymorphism and ovarian cancer risk are shown in Table 2. After Bonferroni correction, we found a close association between XRCC3 rs861539 polymorphism and ovarian cancer risk in two genetic models (GA vs. GG: OR = 0.88, 95%CI = 0.81-0.96, *P* = 0.003; GG vs. GA+AA: OR = 0.89, 95%CI = 0.82-0.96, *P* = 0.004), while no significant association was found in the other three genetic models (G vs. A: OR = 1.07, 95%CI = 1.01-1.13, *P* = 0.018; GG vs. AA: OR = 1.11, 95%CI = 0.99-1.25, *P* = 0.08; GG+GA vs. AA: OR = 1.04, 95%CI = 0.94-1.16, *P* = 0.468).

We implemented subgroup analyses of five genetic models based on ethnicity, control source, and detection method (Table 3). In the ethnicity-based subgroup analysis, XRCC3 rs861539 was associated with a reduced risk of ovarian cancer in Caucasian populations based on the heterozygote model (GA vs. GG: OR = 0.88, 95%CI = 0.81-0.96, *P* = 0.004) and dominant model (GG vs. GA+AA: OR = 0.88, 95%CI = 0.81-0.96, *P* = 0.004). In the subgroup analysis based on control source, hospital-based was significantly increased in the homozygote model (GG vs. AA: OR = 1.30, 95%CI = 1.05-1.62, *P* = 0.016). In subgroup analyses based on detection method, PCR-RFLP was associated with a significant increase in ovarian cancer risk in the homozygote model (GG vs. AA: OR = 1.31, 95%CI = 1.06-1.62, *P* = 0.013).

### 3.3. Publication Bias and Sensitivity Analysis

**Figure 2:**
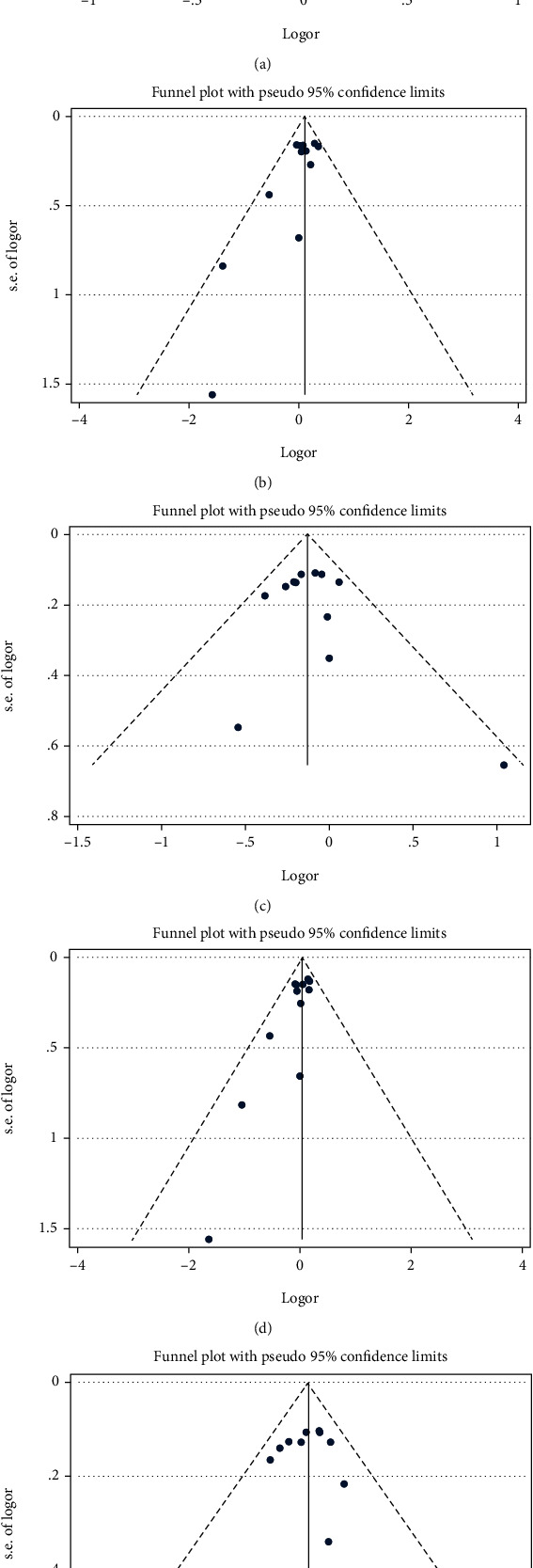
Funnel plots of XRCC3 rs861539 and ovarian cancer risk: (a) G vs. A; (b) GG vs. AA; (c) GA vs. GG; (d) GG+GA vs. AA; (e) GG vs. GA+AA.

**Figure 3:**
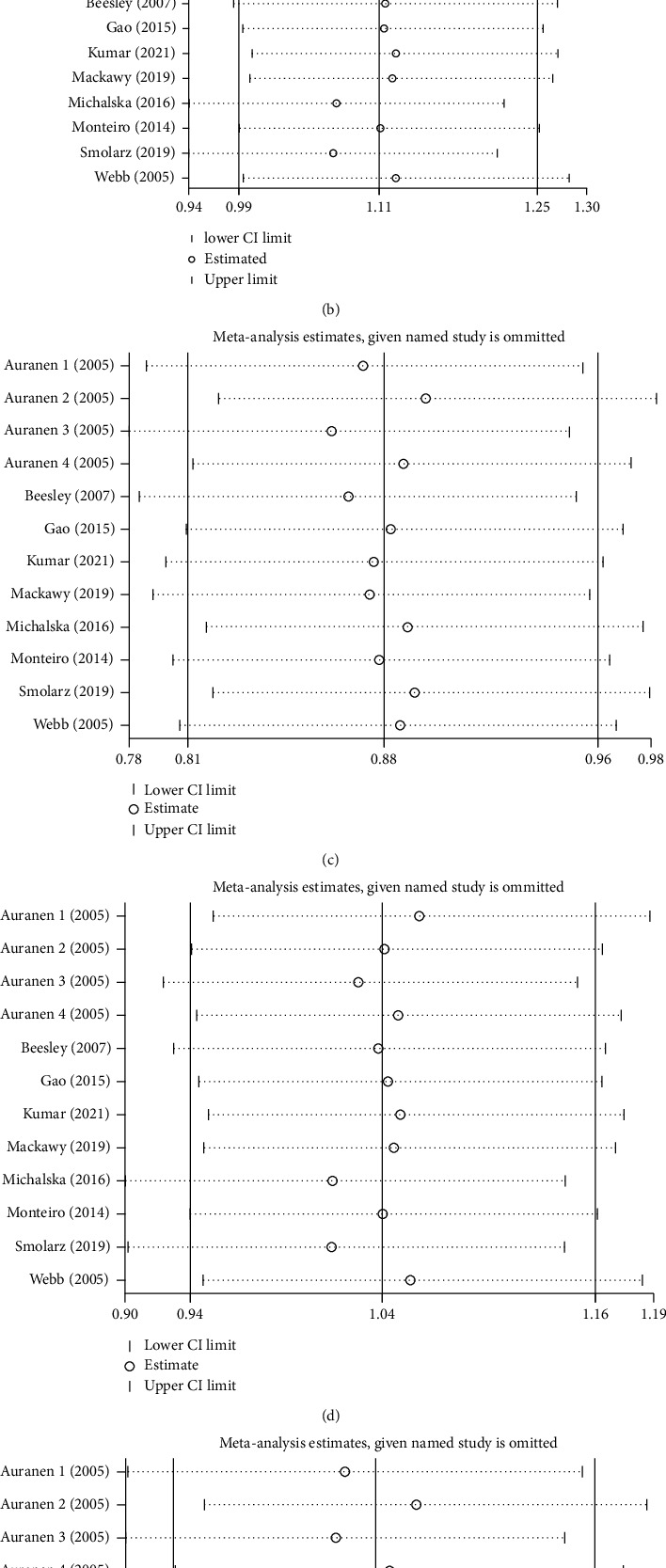
Sensitivity analysis: (a) G vs. A; (b) GG vs. AA; (c) GA vs. GG; (d) GG+GA vs. AA; (e) GG vs. GA+AA.

Funnel plots of XRCC3 rs861539 and ovarian cancer risk are shown in Figure 2. Begg's and Egger's test results are as follows: additive model *P* = 0.732 and 0.087, homozygote model *P* = 0.193 and 0.028, heterozygote model *P* = 0.732 and 0.626, recessive model *P* = 0.064 and 0.012, dominant model: *P* = 0.945 and 0.325. Egger's test *P* < 0.05 in both homozygote and recessive models, indicating publication bias existed in the two models. Sensitivity analysis results illustrated that our results are reliable (Figure 3).

### 3.4. Trial Sequential Analysis

**Figure 4:**
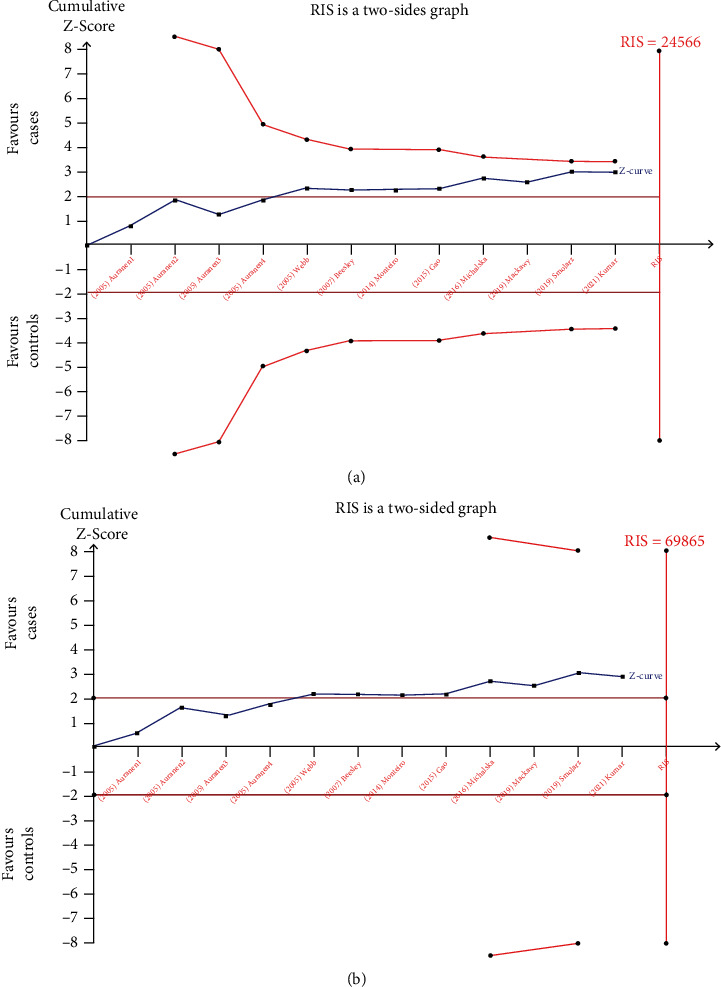
Trial sequential analysis for the meta-analysis of XRCC3 rs861539 and ovarian cancer risk under (a) GA vs. GG and (b) GG vs. GA+AA.

In this meta-analysis, two genetic models produced statistically significant in the estimation of correlation between XRCC3 rs861539 polymorphism and ovarian cancer risk. Therefore, we conducted TSA to analyze the heterozygote model (GA vs. GG) and the dominant model (GG vs. GA+AA). As shown in Figures 4(a) and 4(b), the *Z*-curve crossed the traditional boundary value, but did not cross the TSA boundary value, and the accumulated information did not meet the required information size (RIS), indicating that the meta-analysis may have obtained false-positive conclusions. Therefore, more case-control trials should be included in the future to confirm the conclusions.

## 4. Discussion

In the past few years, the Cancer Genome Project and the Cancer Genome Atlas have intersected with the field of DNA repair, revealing specific characteristics of DNA damage and DNA repair errors in various cancers [5], which has brought more attention to DNA repair genes in recent years. Germline or somatic mutations in HR genes have been found in about one-third of ovarian cancers [4], and approximately 50% of high-grade serous ovarian cancers (HGSOCs) are defective in HR DNA repair pathways [35–37]. Mutations in genes involved in DNA repair, such as BRCA1, BRCA2, RAD51C, RAD51D, BRIP1, BARD1, CHEK2, MRE11A, RAD50, ATM, and TP53, have been found to increase the risk of ovarian cancer [38–40]. As a central role in HR, XRCC3 interacts and stabilizes with Rad51 [9] and plays an important role in preventing genomic instability and the generation of tumorigenic mutations [8]. Shi et al. [41] reported that high XRCC3 expression related to poor OS (Overall Survival) data, suggesting that high XRCC3 mRNA levels might play oncogenic roles in breast cancer. Roos et al. [42] reported that glioma overexpressed XRCC3 compared with normal brain tissue. By downregulating the expression of XRCC3 by interference RNA, it was proved that XRCC3 can protect glioma cells from germ-cell death, apoptosis, and cell cycle inhibition induced by temozolomide. However, Cheng et al. [43] presented that reduced XRCC3 expression leads to increased DNA adducts that contribute to lung tumorigenesis in nonsmokers. Todorovic et al. [44] suggested that the ability of tumor cells to induce an effective DNA damage response immediately after exposure to DNA damage agents promotes drug resistance. Failure to activate DNA repair adequately, on the other hand, can lead to tumor cell death.

A number of studies have reported an association between XRCC3 rs861539 polymorphism and ovarian cancer risk [18–26]. Michalska et al. [21] reported that XRCC3 Thr241Met polymorphism may be positively associated with the incidence of ovarian cancer in Polish women. Smolarz et al. [19] found that homozygotes for the Thr241Met polymorphism reduced ovarian cancer risk. However, the study by Kumar et al. [18] showed that the XRCC3 rs861539 polymorphism was not associated with ovarian cancers in the South Indian population. Therefore, the conclusions of the current research on the relationship between the two are inconsistent. Two previous meta-analyses have explored their relationship. A meta-analysis in 2013 reported the association between Thr241Met polymorphism and ovarian cancer risk and found no significant association [28]. In 2021, a meta-analysis reported no association between the Thr241Met polymorphism and risk of gynecologic malignancies, but found a significantly increased risk of gynecologic malignancies in Asians in an ethnicity-based subgroup analysis. Subgroup analyses were then performed based on tumor type and found no increased risk of ovarian cancer in Asians [27]. This is consistent with our findings on Asians. However, the two meta-analyses did not include all studies related to XRCC3 rs861539 polymorphism and ovarian cancer risk. Therefore, 4,634 ovarian cancer cases and 7,381 controls from 12 studies in 9 articles were included in our meta-analysis.

For the overall data, the current meta-analysis showed an association between XRCC3 rs861539 polymorphism and ovarian cancer risk in the heterozygote model and the dominant model. In the subgroup analysis based on ethnicity, we found that XRCC3 rs861539 was associated with ovarian cancer risk in Caucasians. In addition, after stratifying patients separately according to the control source and detection method, hospital-based studies and PCR-RFLP-based studies were found to be more associated with ovarian cancer risk. In these subgroup analyses, we suspect that ethnicity, control source, and detection method may be the sources of interstudy heterogeneity.

Compared with previous meta-analyses, this meta-analysis included the most comprehensive research with enough samples for in-depth analysis, but it still had some limitations. Firstly, some studies lacked information on subjects' age, age at menopause, and family history, making it impossible to subgroup these factors, which reduces the comprehensiveness of the results. Secondly, many of the relevant studies have been conducted among Caucasians, with fewer studies involving Asians. Thirdly, the interactions of gene-gene and gene-environment may have influenced our results [31, 45]. Fourthly, we searched only Chinese and English databases and may have overlooked some studies published in other languages. Fifthly, the heterogeneities of ethnicity, control source, and detection method all might induce the bias. Finally, as indicated by the TSA results, the inclusion of limited cases and controls reduces the representativeness of the conclusions. Therefore, the conclusion of this meta-analysis needs to be confirmed by more case-control trials.

## 5. Conclusion

In conclusion, the present meta-analysis suggests that the XRCC3 rs861539 polymorphism may be associated with ovarian cancer risk, especially in Caucasians. Considering the limitations of this meta-analysis, large-scale multicenter case-control studies in more different regions will be needed in the future.

## Figures and Tables

**Table 1:** Main characteristics of the studies included in the meta-analysis.

First author	Year	Country	Ethnicity	Cases/controls	Cases			Controls			HWE	Quality score
					GG	GA	AA	GG	GA	AA		
Kumar	2021	India	Caucasian	200/200	136	49	15	140	51	9	0.131	7
Smolarz	2019	Poland	Caucasian	600/600	147	307	146	117	317	166	0.118	8
Mackawy	2019	Egypt	Caucasian	50/20	21	17	12	14	4	2	0.094	7
Michalska	2016	Poland	Caucasian	700/700	180	340	180	150	350	200	0.892	8
Gao	2015	China	Asian	70/70	62	6	2	60	10	0	0.520	8
Monteiro	2014	Brazil	Mixed	70/70	32	33	5	32	33	5	0.368	8
Beesley	2007	Australia	Caucasian	731/747	291	339	101	288	351	108	0.950	7
Webb	2005	Australia	Caucasian	543/1125	229	238	76	438	538	149	0.420	8
Auranen1	2005	UK	Caucasian	749/830	297	347	105	318	404	108	0.248	7
Auranen2	2005	US	Caucasian	270/344	125	114	31	130	174	40	0.111	7
Auranen3	2005	Denmark	Caucasian	361/891	144	168	49	358	394	139	0.080	7
Auranen4	2005	UK	Caucasian	290/1784	130	121	39	728	827	229	0.806	7

**Table 2:** Results of meta-analysis under different genetic models.

Genetic models	*I* ^2^ (%)	Model for analysis	*P*_H_	OR (95% CI)	*P*_Z_
G versus A	9.8	FEM	0.349	1.07 (1.01, 1.13)	0.018
GG versus AA	5.2	FEM	0.395	1.11 (0.99, 1.25)	0.08
GA versus GG	0.0	FEM	0.485	**0.88 (0.81, 0.96)**	**0.003**
GG+GA versus AA	0.0	FEM	0.672	1.04 (0.94, 1.16)	0.468
GG versus GA+AA	13.4	FEM	0.314	**0.89 (0.82, 0.96)**	**0.004**

Bonferroni correction for multiple testing was applied (*P* value threshold 0.01).

**Table 3:** Meta-analysis results of XRCC3 rs861539 polymorphism in different subgroups.

Genetic models	Subgroup	*I* ^2^ (%)	Model for analysis	*P*_H_	OR (95% CI)	*P*_Z_
G versus A	Ethnicity					
	Caucasian	25.7	FEM	0.207	1.07 (1.01, 1.13)	0.017
	Asian	NA	FEM	NA	1.00 (0.40, 2.48)	1.0
	Mixed	NA	FEM	NA	1.00 (0.60, 1.66)	1.0
	Control source					
	Population	0.0	FEM	0.865	1.04 (0.98, 1.12)	0.194
	Hospital	57.3	REM	0.071	1.08 (0.86, 1.35)	0.496
	Detection method					
	TaqMan	0.0	FEM	0.675	1.04 (0.97, 1.12)	0.264
	Sequencing	0.0	FEM	0.909	1.05 (0.91, 1.22)	0.491
	PCR-RFLP	58.4	REM	0.065	1.08 (0.87, 1.33)	0.50
GG versus AA	Ethnicity					
	Caucasian	13.4	FEM	0.319	1.12 (0.99, 1.26)	0.069
	Asian	NA	FEM	NA	0.21 (0.01, 4.39)	0.31
	Mixed	NA	FEM	NA	1.00 (0.26, 3.79)	1.0
	Control source					
	Population	0.0	FEM	0.910	1.04 (0.90, 1.20)	0.603
	Hospital	46.4	FEM	0.133	**1.30 (1.05, 1.62)**	**0.016**
	Detection method					
	TaqMan	0.0	FEM	0.745	1.04 (0.89, 1.22)	0.611
	Sequencing	4.4	FEM	0.306	1.00 (0.73, 1.37)	0.996
	PCR-RFLP	30.9	FEM	0.227	**1.31 (1.06, 1.62)**	**0.013**
GA versus GG	Ethnicity					
	Caucasian	8.2	FEM	0.367	**0.88 (0.81, 0.96)**	**0.004**
	Asian	NA	FEM	NA	0.58 (0.20, 1.70)	0.321
	Mixed	NA	FEM	NA	1.00 (0.50, 1.99)	1.0
	Control source					
	Population	0.0	FEM	0.613	0.90 (0.82, 0.99)	0.029
	Hospital	27.7	FEM	0.246	0.81 (0.67, 0.98)	0.028
	Detection method					
	TaqMan	0.0	FEM	0.420	0.91 (0.81, 1.01)	0.084
	Sequencing	0.0	FEM	0.50	0.83 (0.67, 1.03)	0.097
	PCR-RFLP	26.7	FEM	0.252	0.83 (0.69, 1.00)	0.048
GG+GA versus AA	Ethnicity					
	Caucasian	0.0	FEM	0.607	1.04 (0.94, 1.16)	0.434
	Asian	NA	FEM	NA	5.15 (0.24, 109.15)	0.29
	Mixed	NA	FEM	NA	1.00 (0.28, 3.62)	1.0
	Control source					
	Population	0.0	FEM	0.879	0.98 (0.86, 1.12)	0.815
	Hospital	13.8	FEM	0.323	1.14 (0.96, 1.35)	0.135
	Detection method					
	TaqMan	0.0	FEM	0.709	1.00 (0.86, 1.16)	0.958
	Sequencing	1.3	FEM	0.314	0.92 (0.68, 1.23)	0.568
	PCR-RFLP	0.0	FEM	0.528	1.15 (0.97, 1.36)	0.116
GG versus GA+AA	Ethnicity					
	Caucasian	28.0	FEM	0.187	0.89 (0.82, 0.96)	0.004
	Asian	NA	FEM	NA	0.77 (0.29, 2.09)	0.614
	Mixed	NA	FEM	NA	1.00 (0.51, 1.94)	1.0
	Control source					
	Population	0.0	FEM	0.702	0.91 (0.83, 1.00)	0.05
	Hospital	52.6	REM	0.097	0.86 (0.62, 1.19)	0.358
	Detection method					
	TaqMan	0.0	FEM	0.494	0.92 (0.83, 1.02)	0.114
	Sequencing	0.0	FEM	0.815	0.87 (0.71, 1.07)	0.179
	PCR-RFLP	55.4	REM	0.081	0.89 (0.64, 1.22)	0.462

Bonferroni correction for multiple testing was applied (ethnicity and detection method group: *P* value threshold 0.016; control source group: *P* value threshold 0.025).

## Data Availability

The data analyzed in this study are from previously reported studies, which have been cited.
